# A decade of systematic literature review on Airbnb: the sharing economy from a multiple stakeholder perspective

**DOI:** 10.1016/j.heliyon.2021.e08222

**Published:** 2021-10-19

**Authors:** Sri Rahayu Hijrah Hati, Tengku Ezni Balqiah, Arga Hananto, Elevita Yuliati

**Affiliations:** Management Department Faculty of Economics and Business, Universitas Indonesia, Indonesia

**Keywords:** Airbnb, Literature review, Stakeholder, Guest, Host, Employee, Community, Competitor, Policymaker

## Abstract

Airbnb, which launched its business in 2009, has experienced explosive growth by creating value through the sharing economy business model. The Airbnb business model helps property owners exploit underutilized assets. However, along with its rapid growth, controversies have arisen among many stakeholders, especially the traditional hotel industry, communities, and policymakers. This study reviews academic articles to pinpoint the factors involved in the relationships among Airbnb and its multiple stakeholders. The aim is to identify the benefits, drawbacks, and issues surrounding Airbnb. The analysis is based on the perspectives of six Airbnb stakeholders: guests, hosts, employees, communities, competitors, and policymakers. A variety of scholarly journals indexed in the Scopus database were reviewed, with 282 included in the final analysis. The analysis will be useful for academics, practitioners, and policymakers alike, as it summarizes the Airbnb relevant actors, identifies key factors that influence stakeholder behavior, and assesses the power and level of influence of each stakeholder. Ultimately, the study points to potential directions for future research on Airbnb.

## 1 Introduction

Within a few years of its inception in 2009, Airbnb had become one of the most successful sharing economy platforms. Roelofsen and Minca (2018) report that as of 2017, Airbnb had attracted 100 million hosts and guests worldwide, earning $100 million that year. The company has developed its business model based on a compelling value proposition. It integrates economic benefits for travelers and residents of tourist areas via a trusted marketplace that enables the platform to scale up and leverage its assets through network utilization (Cheng and Foley, 2018; Leoni, 2020; Oskam and Boswijk, 2016).

Airbnb offers many benefits to its stakeholders. For customers, Airbnb accommodation is typically cheaper than traditional accommodation like a hotel (Guttentag, 2015; Gyódi, 2019; Varma et al., 2016). In addition, Airbnb offers local authenticity (Bucher et al., 2018), giving customers the opportunity to live like locals in a listed apartment, house, or private room (Gurran and Phibbs, 2017; Lin et al., 2019; Paulauskaite et al., 2017). For property owners, Airbnb enables them to maximize the utilization of their underutilized assets (Ferreri and Sanyal, 2018; Oskam and Boswijk, 2016). For other stakeholders, such as the community, Airbnb increases community economic and business opportunities (Gurran and Phibbs, 2017; Petruzzi et al., 2020).

Despite the advantages that Airbnb offers, some have been on the receiving end of the negative externalities that Airbnb's growth has brought about. For example, one hotel in Texas experienced a revenue loss for every increase in Airbnb property listings (Dogru et al., 2019; Varma et al., 2016; Zervas et al., 2017). Tourist sites have also been subjected to negative externalities based on the increased concentration of tourists in particular spots, which invites environmental problems such as water scarcity, waste management, and carbon emission issues (Cheng et al., 2020a, Cheng et al., 2020; Martín et al., 2018). The problems created by Airbnb as a shared economy accommodation have also generated challenges for the government as a regulator because Airbnb's disruption of the accommodation industry has changed the tourism landscape, creating taxation problems and discrimination problems, among others (Guttentag, 2015; Interian, 2016; Ključnikov et al., 2018). Many conceptual and empirical studies have discussed these issues from the stakeholders' perspective that include the guests (Pinottti and Moretti, 2018; Sthapit and Björk, 2019), hosts (Ert et al., 2016), competitors (Forgacs and Dimanche, 2016; Ginindza and Tichaawa, 2019; Horodnic et al., 2016), communities (Midgett et al., 2017; Roelofsen and Minca, 2018), and the government (Ključnikov et al., 2018; Schäfer and Braun, 2016).

To date, there are several literature reviews that discuss peer-to-peer (P2P) accommodation in general (Belarmino and Koh, 2020; Dolnicar, 2019, 2020; Prayag and Ozanne, 2018; Sainaghi, 2020) and four literature reviews that address Airbnb specifically (Dann et al., 2019; Guttentag, 2019; Medina-Hernandez et al., 2020; Ozdemir and Turker, 2019).

Our study uses a literature review approach as well. However, unlike the study of Dann et al. (2019)—which discusses the motives of hosts and guests, the role of trust and reputation, price calculation, and legal aspects—our study is based on stakeholder theory. We structure our study around this theory as it brings an ethical aspect to management decision making (Goodpaster, 1991). The ethical issue is relevant as ethics is a theme that remains underexplored in the research related to the sharing economy (Andreu et al., 2020).

Based on stakeholder theory, as suggested by Reed et al. (2009), our study is framed around three questions: (1) who are the stakeholders of Airbnb? (2) what are their interests/concerns? and (3) how much power and influence does each stakeholder have? We expect our results to generate knowledge about the relevant Airbnb actors and to provide a comprehensive understanding of the Airbnb phenomenon, identifying key factors that influence the behavior of its stakeholders, assessing the influence and level of impact of each stakeholder, and exploring the research based on the perspective of Airbnb. As a business entity, Airbnb can use the information to improve stakeholder decision making that incorporates ethical considerations. Further, the study can offer insights to government when considering the feasibility of future policy directions.

## 2 Literature review

### 2.1 The sharing economy of Airbnb

The sharing economy is sometimes called the “collaborative economy” (Calo and Rosenblat, 2017). The term refers to online network-based activities that provide temporary access to a good to facilitate more efficient use of physical assets. It depends on trust and the capability of operating at a near-zero marginal cost (Frenken et al., 2015; Hawlitschek et al., 2018; Ranjbari et al., 2018; Uzunca and Borlenghi, 2019). Gerwe and Silva (2020) generalize the definition of the sharing economy to be a socioeconomic system that allows peers to grant temporary access to underutilized physical and human assets via an online platform. This socioeconomic system definition captures that the sharing economy can cover both fee-based and non-fee-based transactions (Gerwe and Silva, 2020). The sharing economy offers both advantages and disadvantages to its stakeholders. As a platform, it provides greater flexibility, fair compensation, match-making, an extended reach, trust building, and collectivity in the sharing and collaborative exchange among actors (Hawlitschek et al., 2018; Sutherland and Jarrahi, 2018). Some scholars also see the sharing economy as a wealth redistribution method (Rifkin, 2014) with some benefits for society. Despite being acknowledged as an innovation that decentralizes and disrupts the existing socio-technical and economic regimes, the sharing economy may be accused of being a "neoliberal system on steroids" as well (Martin, 2016; Murillo et al., 2017).

In the hospitality industry, Servas International pioneered the concept of the sharing economy in 1994, followed by CouchSurfing in 2003, and Airbnb in August 2008 (Gerwe and Silva, 2020; Johnson and Neuhofer, 2017). Couchsurfing represents a non-fee-based transaction, while Airbnb represents a fee-based transaction in the sharing economy (Gerwe and Silva, 2020). However, among these three shared accommodation platforms, Airbnb has been the most successful, as it offers a more distinctive service quality and a more local experience than traditional accommodations do (Mody et al., 2017; Varma et al., 2016).

Since its inception more than a decade ago, Airbnb has experienced explosive growth. As of April 2019, Airbnb was available in more than 1,000 cities across the world and was expected to have served more than 500 million guests (Airbnb, 2019a). Airbnb has also gained public trust, reflected in the 250 million reviews it has received from both guests and hosts as of 2019 (Airbnb, 2019a). In 2018, Airbnb had a market valuation of nearly $31 billion, $2.6 billion in profit, and $93 million in revenue (Bloomberg, 2019).

### 2.2 Stakeholder analysis

Donaldson and Preston (1995, p. 67) define stakeholders as “persons or groups with legitimate interests in procedural and substantive aspects of corporate activity. Stakeholders are recognized by their interests in the corporation, whether the corporation has any related functional interest in them.” According to these authors, a group is qualified as a stakeholder if it has a legitimate interest in an aspect of the organization's activities. Such groups can consist of customers, employees, investors, suppliers, communities, governments, trade associations, and political groups. Thus, stakeholders are those who have the power to affect organizational performance and/or have a stake in the organization's performance (Reed et al., 2009). Without the support of stakeholders, a company could not exist.

Stakeholder theory addresses business ethics, morals, and values when managing stakeholders involved with a project or organization (Freeman et al., 2004). The theory is beneficial for any platform-based business in managing synergies and cooperation among its stakeholders to capture value and maintain business sustainability (Laczko et al., 2019). However, it is worth noting that given the different stakeholder interests in a business, it is inescapable that conflicts will arise among them (Harrison and Wicks, 2013). By analyzing how each stakeholder is positively and/or negatively impacted by the organization, the organization can gain alignment among all stakeholder goals and address conflicts early on to create better value for the range of its constituents (Freeman et al., 2012).

### 2.3 Literature review on Airbnb

A literature review is a comprehensive summary and critical analysis of existing relevant academic and non-academic literature on the topic under review, conducted as objectively as possible (Cronin et al., 2008; Linde and Willich, 2003; Rowley and Slack, 2004). Scholars conduct literature reviews to encapsulate the research, critically assess the contributions, and clarify any alternative views in the studies (Rowe, 2014).

In the context of Airbnb, there are four review articles that discuss the platform specifically (Dann et al., 2019; Guttentag, 2019; Medina-Hernandez et al., 2020; Ozdemir and Turker, 2019). The review by Dann et al. (2019), discusses the motives of both hosts and guests, the role of trust and reputation, Airbnb price calculations, the impact of Airbnb on the hotel industry and housing market, and legal and regulatory aspects surrounding Airbnb. The review by Ozdemir and Turker (2019) discusses Airbnb from the perspectives of academics and journalists. Their article discusses legal, social, and economic issues, as well as public relations and publicity, benefits, impacts on destinations, hotel competition, the nature of the sharing economy, technology, consumer behavior, sustainability, corporate social responsibility (CSR), safety and security, growth, politics, and insurance. The intention of the review by Medina-Hernandez et al. (2020) on Airbnb is to illustrate the scarcity of research on P2P accommodation platforms other than Airbnb. Our literature review of Airbnb differs from the four previous reviews through our application of stakeholder theory to our analysis of the Airbnb phenomenon and its embedded ethical perspective. As discussed earlier, the sharing economy, including Airbnb, offers both benefits and drawbacks to its stakeholder since its been successfully reframed by regime actors as an economic opportunity (Martin, 2016). Therefore, stakeholder theory, which introduces ethical issues into management decision making (Goodpaster, 1991), is useful in evaluating whether ethically, Airbnb has been treating its stakeholders interests as equally important and deserving of joint “maximization.”

## 3 Research method

A systematic literature review identifies, evaluates, and interprets all the existing research relevant to a particular research question, topic area, or phenomenon of interest (Kitchenham, 2004). It is important to note that systematic reviews differ from traditional reviews, which are usually conducted with no protocol, no search strategy, and no well-defined methods (Knoll et al., 2018). Our study uses a systematic review with an a priori protocol and a well-defined search strategy and research method. The benefit of a systematic literature review is that it summarizes existing research, identifies the conceptual content, and contributes to theory development (Zhu and Sarkis, 2016).

### 3.1 Initial search

We used the Scopus database to ensure comprehensive coverage of the social scientific journals. We chose Scopus as this database outperforms others, such as Web of Science, in terms of journal coverage and the number of documents retrieved, especially in the field of social science (Aksnes and Sivertsen, 2019). The time window for the article search was from January 1, 2009 until July 8, 2020.

The keywords used for data collection were “Airbnb,” “customer,” “consumer,” “guest,” “host,” “employee,” “community,” “hotel,” “competitor,” “community,” “city,” “government,” “policy maker,” AND “regulator.” We used several combinations of these keywords, including (1) Airbnb AND stakeholder, (2) Airbnb AND customer, (3) Airbnb AND consumer, (4) Airbnb AND guest, (5) Airbnb AND host, (6) Airbnb AND employee, (7) Airbnb AND hotel, (8) Airbnb AND competitor, (9) Airbnb AND community, (10) Airbnb AND city, (11) Airbnb AND government, (12) Airbnb AND policy maker, and (13) Airbnb AND regulator. The initial search resulted in a total of 1418 papers (see Table 1).

**Table 1:** Initial search results.

Search keywords	Search results
Airbnb AND stakeholder	37
Airbnb AND customer	111
Airbnb AND consumer	158
Airbnb AND client	6
Airbnb AND guest	172
Airbnb AND host	275
Airbnb AND employee	10
Airbnb AND competitor	21
Airbnb AND hotel	156
Airbnb AND hostel	2
Airbnb AND inn	2
Airbnb AND motel	3
Airbnb AND resort	2
Airbnb AND “bed and breakfast”	7
Airbnb AND lodging	57
Airbnb AND community	90
Airbnb AND city	221
Airbnb AND neighbors	10
Airbnb AND government	42
Airbnb AND policy maker	14
Airbnb AND regulator	13
Airbnb AND authority	9
Grand Total	1418

### 3.2 Filtering

As many papers appeared in more than one category, eliminating these duplications left us with 944 documents. Among these, were journal articles (633), conference papers (203), book chapters (31), reviews (28), notes (11), letters (10), conference reviews (9), books (7), short surveys (6), editorials (2), errata (2), and undefined (2). All the non-article papers were excluded, resulted in 633 papers. We only included journal articles, since these have a greater credibility than other content due to the peer review process. The peer review process for journal articles submitted by scholars, means that they are evaluated by experts in the field before publication (The Open University, 2020). We limited the papers to peer-reviewed journal articles only, leaving us with 630 papers. The results were then further limited to English language articles only, resulting in 610 documents. Finally, we screened for papers that specifically discussed Airbnb's stakeholders in depth, which generated 347 documents. The articles that did not discuss Airbnb relationships with stakeholders or discussed Airbnb in different contexts were eliminated; for example, the macroeconomic environment of Airbnb (Heo and Blengini, 2019). To reduce the potential bias that comes from a single researcher doing all data collection, and to enhance the quality and credibility of the study, we applied analyst triangulation (Patton, 1999), involving four researchers in every stage of the literature review process and analysis.

Each article identified in the literature search was given an initial classification according to the type of stakeholder mentioned in the text (e.g., host, guest, competitor). Later, each article was read in detail for further verification and a more specific classification of themes/categories within each stakeholder discourse. After we read through the abstracts and content of the articles, we had a final sample of 282 articles.

**Figure 1:**
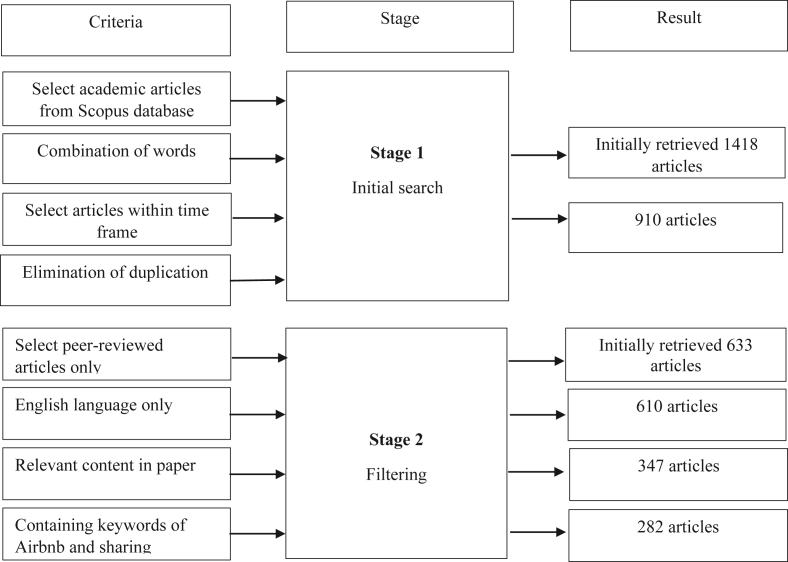
Literature review process.

Our systematic literature review method is shown in Figure 1.

## 4 Analysis

### 4.1 General features of the literature

Since Airbnb was established just over 10 years ago in 2009, a lag in peer-reviewed journal publications is understandable. It takes time for scholars to conduct research, analyze data, and write and submit articles. In addition, the publication process in a peer-reviewed journal can take 18 months in the fields of business and economics (Björk and Solomon, 2013). Thus, it is reasonable that articles on topics related to Airbnb and its stakeholders published in Scopus have appeared only since 2015 (see Figure 1). Indeed, there has been consistent growth in publications related to Airbnb in peer-reviewed journals over the last four years (2017–2020). The highest number of articles on Airbnb was in 2019 (108 articles).

**Figure 3:**
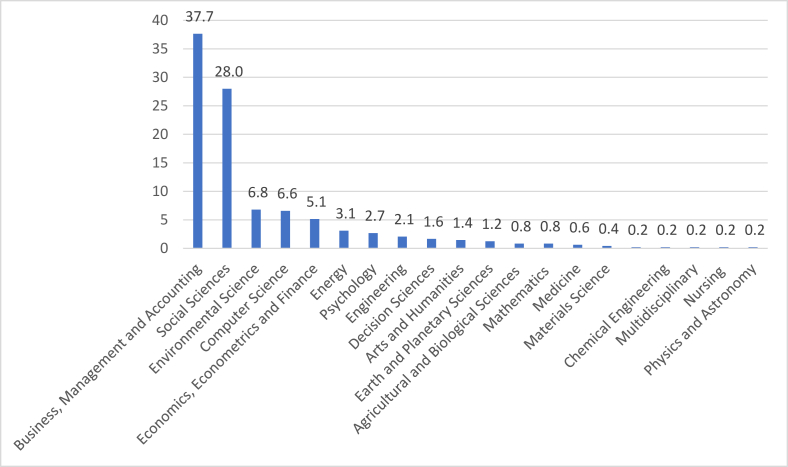
Number of academic publications on Airbnb by discipline.

Based on the subject, two main fields dominated the content: the business, management, accounting subject area (37.7%) and the social sciences subject area (28%). The results indicate that the research on Airbnb—as represented by the Scopus database—focuses generally on the business and social aspects of Airbnb as part of the sharing economy accommodation (see Figure 3).

### 4.2 Who are the Airbnb stakeholders?

**Figure 2:**
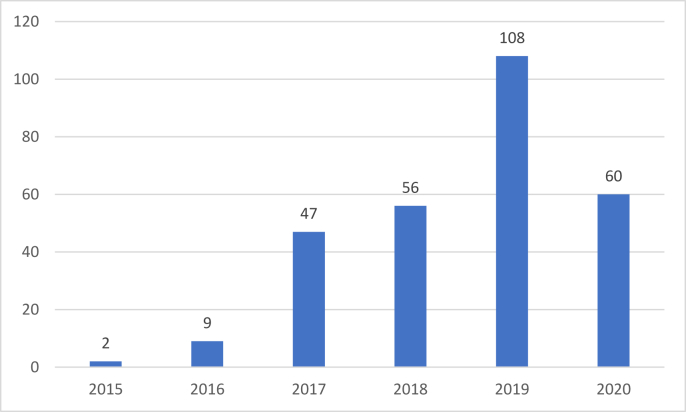
Number of academic articles on Airbnb by year.

Based on its official website, Airbnb considers hosts, guests, employees, and communities as its main stakeholders (Airbnb, 2019a, Airbnb, 2019b) (see Table 2). However, other sources also consider hotels, other platforms similar to Airbnb, and government or regulators as parties influenced by the existence of Airbnb (Cheng and Foley, 2018). The search results confirm these results. Based. our review, most articles on Airbnb published in peer-reviewed journals discuss guests or customers of Airbnb (93 articles; 32%), followed by hosts (88 articles; 30%), communities (56 articles; 19%), competitors (32 articles; 11%), government (24 articles; 8%), and employees (0 articles; 0%). We reviewed a total of 288 articles, but 26 discuss more than one stakeholder; for example, Forgacs and Dimanche (2016) discuss three stakeholders: hosts, the government, and communities. We also found a common overlap between community and competitor stakeholder groups. Thus, according to the stakeholder category, we reviewed a total of 293 articles (see Figure 2).

**Table 2:** Number of articles published on Airbnb by stakeholders.

Stakeholders	Total	Percentage
Host	88	30
Guest	93	32
Community	56	19
Competitor	32	11
Employee	0	-
Government	24	8
Total	293	100

The following section discusses Airbnb from the perspective of these stakeholders: (1) hosts, (2) guests, (3) employees, (4) communities, (5) competitors, such as traditional hotels and other platforms similar to Airbnb, and (6) government/regulators.

### 4.3 What are the Airbnb stakeholders’ interests/concerns?

#### 4.3.1 Hosts

Johnson and Neuhofer (2017) suggest that hosts play an important role as the primary social contact starting from the familiarization pre-stay stage and, most importantly, to aid in value co-creation. We found that studies on Airbnb hosts cover a relatively broad sub-topic. Table 3 indicates the general categories of papers identified in the literature search. Out of 282 articles, 88 are related to hosts. We classified the host literature into 11 themes (see Table 3).

a. Decision to take guests

**Table 3:** Variables related to Airbnb and host relationships.

Category	Independent Variable	Dependent Variable	Author
a. Host visual image	Trust	Price	Ert et al. (2016); Ert and Fleischer (2019)
	Facial expression	Likelihood to rent	Fagerstrøm et al. (2018, 2017)
	Facial expression	Avoidance behavior	Fagerstrøm et al. (2017)
	Beauty/attractiveness	Rental price	Jaeger et al. (2018)
	Smile intensity	Price	Jaeger et al. (2018)
	Absence of host profile	Likelihood to rent	Fagerstrøm et al. (2017)
b. Decision to take guests	Guest with profile picture	Decision to take a guest	Karlsson et al. (2017)
	Positive self-reference		
	Female guest		
	Older guest		
	couples		
	Single		
	Length of stay		
	Purpose of trip		
c. Host status and reviews	Host status	Price	Abrate and Viglia (2019); Benítez-Aurioles (2018a); Cai et al. (2019); Contu et al. (2019a); Ert and Fleischer (2019); Leoni and Parker (2019); Wang and Nicolau (2017)
		Likelihood to rent	
		Property owner profit	
	Rating score	Price	
	Duration of membership	Price	
	ID verification	Price	
	Host reputation	Revenue	
	Number of reviews	Probability to be a superhost	
	Review sentiment score	Rental room price	Lawani et al. (2019)
	Price and superhost status	Review volume	Liang et al. (2017)
	Personal reputation and product description	Rating	
	Hosts' service attributes	Popularity (e.g., ratings, number of reviews, superhost status)	Mauri et al. (2018)
	ID verification, account review, and superhost status	Superhost status Platform control over hosts	Contu et al. (2019a); Sun et al. (2019) Leoni and Parker (2019)
	Cultural factors, trust, reciprocity, lack of anonymity, Airbnb policy	Positive ratings	Bridges and Vásquez (2018)
d. How hosts market their listings	Use of social words in listing description Place picture	Revenue	Han et al. (2019); Nieto García et al. (2019)
	Host race	Listing description	Törnberg and Chiappini (2020)
	Host self-presentation	Guest response to self-presentation strategy Trust	Tussyadiah and Park (2018) Zarifis et al. (2019)
	Host language style in listing profile	Guest segmentation and targeting Number of guest reviews	Lutz and Newlands (2018) Fierro and Aranburu (2018)
	Host linguistic and semantic features in self-description	Trust	Zhang et al. (2020)
e. Host motivations	Economic opportunities Social benefits Macro-level determinants National cultural values	The decision to host on Airbnb Host-guest interaction level	Dogru et al. (2020c); Farmaki and Stergiou (2019); Gerwe et al. (2020); Gupta et al. (2019); Zhang et al., 2019a, Zhang et al., 2019b
	Perceived risks Economic opportunities, Personal circumstances	Continuance intention to host Marginal hosts' decision to host on Airbnb	Malazizi et al. (2018) Semi and Tonetta (2020)
	Forms of benefits derived from vacations and vacation alternatives	-	Dolnicar and Talebi (2020)
f. Host–guest interactions	Host practices Relationship marketing practices Spatial triad dimensions Perception of space Power dynamics	Value co-creation/Value co-destruction/value recovery Host-guest interactions	Belarmino et al. (2019); Camilleri and Neuhofer (2017a); Casais et al. (2020); Farmaki et al. (2020); Farmaki and Christou (2019); Farmaki and Kaniadakis (2020); Johnson and Neuhofer (2017); Tussyadiah and Zach (2017)
	Role of hosts, home, and platform	Host–guest talk and interaction	Scerri and Presbury (2020)
	Host unpleasant behaviors	Distrust in the host Value co-destruction	Sthapit (2019); Sthapit and Björk (2019)
	Host's social interactions and attitude	Trust and positive Airbnb experience	Sthapit and Jiménez-Barreto (2018)
	Sense of community	Trust	Huurne et al. (2020)
	Forms of discrimination by host Reasons for discrimination	Host's discrimination practice	Farmaki and Kladou (2020)
	Host experience with guests from a different culture	Host–guest interactions	Cheng and Zhang (2019)
g. Host–platform and host-community relationships	Attachment and psychological ownership toward the platform	Organizational citizenship behavior toward Airbnb	Lee et al. (2019b)
	Social antecedents, technical antecedents, economic antecedents, privacy assurance antecedents	Hosts trust toward Airbnb	Wang et al. (2020)
	Existence of algorithmic controls	Host reactions toward algorithmic control	Cheng and Foley (2019)
	Role of Airbnb host community	Risk mitigation Learning	Ravenelle (2020) Cheng et al. (2020b); Holikatti et al. (2019)
h. Host perceptions	Perceptions of host's moral responsibilities	Behaviors that reflect moral responsibilities.	Farmaki et al. (2019)
	Perception of Airbnb environmental certification program Motivations toward participating in Airbnb environmental certification program	Willingness to participate in an environmental certification program.	Fudurich and MacKay (2020)
	Perceived difference in standards, focus on people and places, host-guest dynamics	Why some people host in Airbnb but not in Couchsurfing and vice versa.	Klein et al. (2017)
	Perceptions toward impaired guests	-	Randle and Dolnicar (2019)
i. Host demographics	Gender	Likelihood to rent	Fagerstrøm et al. (2017)
	Race	Price	Jaeger et al. (2018); Kakar et al. (2018); Marchenko (2019)
	Income, education	Participation in Airbnb	Ke (2017)
j. Host prices and performance	Types of listings offered Number of listing managed by a host	Variation in Airbnb pricing applied by hosts	Gibbs et al. (2018b)
	Host experience	Listing revenue performance	Koh et al. (2019); Kwok and Xie (2019); Oskam et al. (2018)
	Price positioning, dynamic pricing, number of listings managed	Price	Gibbs et al. (2018a); Lawani et al. (2019); Moreno-Izquierdo et al. (2019); Önder et al. (2019); Voltes-Dorta and Inchausti-Sintes (2020); Zhang et al. (2017)
	Physical characteristics Host characteristics, location, and number of reviews	Price	Lorde et al. (2019)
	Site (property) reputation, convenience, personnel, and amenities	Price	Magno et al. (2018)
	Host accumulated experience and level of market demand	Price	Proserpio et al. (2018)
	Reciprocity and rating	Price	Ram and Hall (2018)
	Walkability	Number of reviews	Tang et al. (2019)
	Site characteristics and situational characteristics	Price	Wang and Nicolau (2017)
	Host attributes, site and property attributes, amenities and services, rental rules, and online review ratings	Price	
	Host quality, listing characteristics	Listing performance	Xie and Mao (2017)
	Locational strategy	Revenue performance	Xie and Mao (2019); Xie et al. (2020); Zekan et al. (2018)
	Functional attributes, signal attributes (price, market, host-provided signal, platform-provided signal)	Probability of listing being booked	Yao et al. (2019)

The second theme relates to the host's decision to take guests. The study by Karlsson et al. (2017) carries out a choice experiment with 192 Airbnb hosts in Australia. They find that hosts prefer to grant permission to guests who provide profile pictures, have a positive self-reference, are older in age, female, are couples or who are portrayed alone in the picture. In addition to a guest profile, the host's preference is also affected by trip characteristics, such as length of booking (hosts prefer longer booking), the purpose of the trip (e.g., having a holiday is preferred to celebrating one's birthday).

b. Host status and reviews

The third theme is about host status and reviews. Host status and reputation (e.g., superhost status, rating score, number of reviews, review sentiments, etc.) also affect many dependent variables such as revenue, price, likelihood to rent, and property owner's profit (Benítez-Aurioles, 2018a; Cai et al., 2019; Ert and Fleischer, 2019; Lawani et al., 2019; Wang and Nicolau, 2017).

Superhosts are hosts who have 10 completed visits from at least 80% of their guests, a response rate of at least 90% to booking inquiries, and a low cancellation rate in the last 12 months (Gunter, 2018). Benítez-Aurioles (2018a) and Liang et al. (2019) find that a superhost badge has a positive relationship with rent prices listed in Airbnb, indicating that hosts with superhost status can command higher rents from their guests. Besides affecting price and revenue, host status also influences other variables, such as review volume, rating, and popularity (e.g., Liang et al., 2017; Mauri et al., 2018). Host status depends on ratings. Notably, the tendency of guests to give positive ratings to Airbnb hosts is influenced by a culture of politeness, trust between host and guest, review and rating reciprocity, lack of anonymity, and the removal of reviews that violate Airbnb's guidelines (Bridges and Vásquez, 2018).

Based on a data set of more than 40,000 Airbnb listings from San Francisco and the Bay Area aggregated for the period between September 2014 and August 2016, Gunter (2018) reports the ranking of four criteria needed to be a superhost: an excellent rating, reliable cancellation behavior, responsiveness, and sufficient Airbnb demand. This corroborates the findings of Leoni and Parker (2019), who suggest that Airbnb uses ratings (in addition to reviews) as a measure of platform performance and platform owner profit, which, at the same time, serves as a control mechanism to align host and platform objectives. However, the impact of host status on listing price is not always consistent; while the average rating score and duration of membership significantly influence the listing price, the impact of superhost status and identification verification on price is not consistent (Wang and Nicolau, 2017).

c. How hosts market listings

The fourth theme revolves around how hosts market their listings. The literature offers insights about the way hosts market their listings on Airbnb using their profiles and listing descriptions (Fierro and Aranburu, 2018; Han et al., 2019; Lutz and Newlands, 2018; Nieto García et al., 2019; Törnberg and Chiappini, 2020; Tussyadiah and Park, 2018; Zarifis et al., 2019; Zhang et al., 2020). The literature reveals that hosts are encouraged to use social words in their listing descriptions to achieve higher revenue (Han et al., 2019; Nieto García et al., 2019). Specifically, hosts are advised to adjust their language style to reflect the market segment they wish to target (Lutz and Newlands, 2018). This approach is expected to have a positive effect on trust (Zarifis et al., 2019) and the number of reviews (Fierro and Aranburu, 2018). Similarly, the way in which hosts describe themselves in their profiles can affect guests’ reactions to their profiles (Tussyadiah and Park, 2018; Zhang et al., 2020). Hosts are reminded that Airbnb erases the boundaries between private and economic spheres, compelling disclosure of personal and sometimes intimate information about themselves (Teubner and Flath, 2019).

d. Host motivations

The fifth central theme in the literature is about the motivation to become an Airbnb host. This involves the following benefits: for example, economic, social, cultural, and technical advantages, complemented by some macro-level factors such as tourism demand, wages, and unemployment (e.g., Dolnicar and Talebi, 2020; Farmaki and Stergiou, 2019; Gerwe et al., 2020; Gupta et al., 2019; Malazizi et al., 2018; Semi and Tonetta, 2020; Zhang et al., 2019a).

e. Host–guest interactions

The dynamics of host–guest interactions that help co-create or co-destroy value is the sixth theme in the literature. For instance, an Airbnb host's local knowledge, tips, social experience, and interactions can add value for guests (Belarmino et al., 2019; Camilleri and Neuhofer, 2017a; Zervas et al., 2017). In addition, host behaviors and social interactions with guests (e.g., helpfulness, responsiveness, and communication friendliness and social interactions, efforts to accommodate guests' needs, leaving gifts, etc.) can contribute to value co-creation and promote trust (Casais et al., 2020; Farmaki et al., 2020; Farmaki and Kaniadakis, 2020; Johnson and Neuhofer, 2017; Scerri and Presbury, 2020; Sthapit and Jiménez-Barreto, 2018; Tussyadiah and Zach, 2017). However, it is important to note that for the customers, social interaction is only a secondary factor to basic functionality (Chen and Xie, 2017). By contrast, undesirable behaviors, unresponsive communication, and discrimination practices, and differences in customs and expectations between hosts–guests of different cultures can contribute to value co-destruction, for example, tension creation (Camilleri and Neuhofer, 2017a; Cheng and Zhang, 2019; Farmaki et al., 2020; Farmaki and Kladou, 2020; Huurne et al., 2020; Sthapit, 2019; Sthapit and Björk, 2019).

f. Host–platform and host–community relationships

The seventh and eighth themes revolve around host relationships with the Airbnb platform and the relationship between fellow hosts in the Airbnb host community. One of the characteristics of the sharing economy platform is the use of the platform infrastructure and policies, such as algorithmic controls (Cheng and Foley, 2019), which can affect hosts’ trust (Wang et al., 2020) and organizational citizenship behavior toward Airbnb (Lee et al., 2019a, Lee et al., 2019b). To cope with the tight platform controls and the expectations of wanting to attain high status, positive reviews, and reputation, the hosts make use of the Airbnb online host community for risk mitigation (Ravenelle, 2020). The host community can help them learn how to become a better host from other experienced hosts (Cheng et al., 2020b; Holikatti et al., 2019).

g. Host perceptions

The variety of host perceptions related to Airbnb is the ninth theme. These studies discuss the perceptions of hosts toward guests with disabilities (Randle and Dolnicar, 2019), the host's moral responsibilities (Farmaki et al., 2019), perceptions of the Airbnb environmental certification program (Fudurich and MacKay, 2020), and the perceived differences between Airbnb and Couchsurfing (Klein et al., 2017).

h. Host demographics

The tenth theme relates to host demographics, for example, gender. Fagerstrøm et al. (2018) find that a male host's smiling expression can be regarded as more favorable than a female host's smiling expression, while anger or a neutral facial expression displayed by a male host might invoke a stronger negative influence than the same by a female.

i. Host's pricing and performance

The final theme identified in the literature is host pricing and the performance of listings. The literature suggests several variables affect the pricing and performance of an Airbnb listing, such as its physical characteristics and amenities, the host's characteristics, the host's reputation, its location and distance from points of interest, its ratings and the number of reviews, market demand, situation, price positioning, dynamic pricing, and the number of listings managed by the host (Gibbs et al., 2018a, 2018b; Kwok and Xie, 2019; Lawani et al., 2019; Lorde et al., 2019; Magno et al., 2018; Moreno-Izquierdo et al., 2019; Önder et al., 2019; Oskam et al., 2018; Proserpio et al., 2018; Ram and Hall, 2018; Tang et al., 2019; Voltes-Dorta and Inchausti-Sintes, 2020; Wang and Nicolau, 2017; Xie and Mao, 2019; Xie et al., 2020; Yao et al., 2019; Zekan et al., 2018; Zhang et al., 2017).

#### 4.3.2 Guests

Based on the literature review, the factors that influence guests’ intention, repurchase intention, satisfaction, and loyalty are as follows.

a. Purchase intention

Several researchers investigate the predictors of the intention to use Airbnb services. These studies find that customers' purchase intention for sharing accommodation services, such as Airbnb, is significantly influenced by trust (Pung et al., 2019; Ye et al., 2019). Consumers consider Airbnb a service with a sense of human contact, personalness, sociability, human warmth, and human sensitivity through its P2P platform. This affects customers' perception of its usefulness, ease of use, and its ability to offer social connectedness to local residents, which, in the end, creates high trust in Airbnb (Ye et al., 2019). From the negative perspective, distrust and insecurity influence a customer's intention to use Airbnb as well (So et al., 2018).

Pappas (2017) conducts research that configures the relationships among several variables: trust, benefits, social risk, and economic aspects. His study shows that despite the riskiness of online transactions, consumers still feel that the benefits of Airbnb outweigh the risks. Therefore, they have sufficient purchase intention. By contrast, Amaro et al. (2018) find that perceived risk does not influence Millennials' intentions to book on Airbnb; instead, subjective norms, desires for unique accommodation, attitudes, and economic benefits significantly affect Millennials’ intentions (Amaro et al., 2018). Similarly, in China and India, the antecedents of intention, as described in the Theory of Planned Behavior, all significantly influence customer intention to book on Airbnb (Ma et al., 2020) (Chatterjee et al., 2019).

In addition, Pappas (2017) finds a configuration between social and economic aspects. His study reports that the concept of monetary value is essential for tourists, especially during an economic recession. Airbnb, in this context, can provide affordable solutions for consumers. However, consumers are willing to pay a premium price for an Airbnb if they perceive that the interaction among customers and the host is highly functional, emotional, and offers social value (Zhang et al., 2018).

Mody et al. (2017) find that the experience's memorability directly influences customers' behavioral intention to use Airbnb. The memorability of the experience is generated from an extraordinary outcome consisting of meaningfulness (e.g., a renewed sense of self) and well-being (e.g., enhanced quality of life). The memorability of the experience is the product of the experience economy, which consists of entertainment, escapism, education, aesthetics, serendipity, localness, community, and personalization (Mody et al., 2017). It is important to note that poor accommodations and service or hosts' unpleasant behaviors create a negative memorable experience for the Airbnb guest (Sthapit et al., 2020).

Another factor influencing customers to use Airbnb are its ratings. Although rating volume has an insignificant impact on perceived value, it significantly affects purchase intention (Chen and Chang, 2018). In addition, information quality has a positive and significant impact on satisfaction, thereby affecting purchase intention (Chen and Chang, 2018). Another study shows that hedonic motivation, price value, and habit have positive effects on behavioral intention (Lin et al., 2017). One study supporting these results shows the significant impact of pricing sensitivity on perceived value and purchase (Liang et al., 2018). Purchase intention is also influenced by perceived risk, perceived authenticity, and electronic word-of-mouth (e-WOM) (Liang et al., 2018). The level of guest involvement (Lee and Kim, 2018), Airbnb's reputation, guests' unique experience, and guests' attachment also influence intention to stay in an Airbnb (Tiamiyu et al., 2020).

Other factors mentioned that may drive purchase intention are advertising appeal, the individual's sense of power, and self-brand connection (Liu and Mattila, 2017). However, it is important to note that factors that motivate customers to use an Airbnb differ from those that demotivate them to use it. A study by Tran and Filimonau (2020) in Vietnam identifies economic value, functional attributes, and location as affecting purchase intentions, while the lack of safety and unfamiliarity are the demotivators of Airbnb purchase intention.

In the sharing economy, the service users and the service providers form a two-sided market around the platform. Sung et al. (2018) conclude that the consumer model and provider model should be structured separately to gain a better understanding of the business. For consumers, enjoyment and the network effect influence consumer attitude and intention to become Airbnb guests. For providers, economic benefit, sustainability, social relationships, and the network effect influence their attitudes and intention to become Airbnb hosts. The network effect is the only factor that shows significance in both the consumer and the provider models.

b. Repurchase intention

Liang et al. (2018) state that repurchase intention is influenced by trust in the company (Airbnb), trust in the hosts, and transaction-based satisfaction. Another study concludes that for Airbnb consumers, intention to repurchase is influenced significantly by attitudes and subjective norms, whereas perceived behavioral control is not (Mao and Lyu, 2017). Perceived value and risk have significant direct impacts on attitude and, in turn, indirectly affect repurchase intention. Furthermore, Mao and Lyu's (2017) research finds that unique experience expectations, familiarity, and e-WOM influence repurchase intention. A study conducted in the US shows that customer experience comprises four dimensions of home benefits, personalized services, authenticity, and social connection, are significant predictors for Airbnb users' behavioral intention (Li et al., 2019). A study by Liang et al. (2020) reveals how guests' booking behavior is influenced by host-generated content associated with the amount of information and topics.

Wang and Jeong (2018) also conclude that attitudes and satisfaction are both significant predictors of Airbnb customers' intention to choose Airbnb again. Amenities and host-guest relationships are substantial predictors of guest satisfaction, while perceived usefulness and trust positively affect Airbnb's customer attitudes.

Customer satisfaction is the last predictor of customer's repurchase intention mentioned (Birinci et al., 2018). Customer satisfaction itself is significantly influenced by perceived authenticity and safety/security risk factors. In other words, Airbnb customers feel satisfied if they experience ways to live and interact with the local community. In addition, customers feel satisfied with Airbnb based on their transactions and the personal information provided (Birinci et al., 2018).

c. Satisfaction

Concerning satisfaction, the two-factor theory can be applied to explain the Airbnb phenomenon. The facilities of Airbnb are considered a hygiene factor, while the home experience and host attitude are the other motivating factor for tourist satisfaction (Xu et al., 2019a, Xu et al., 2019b).

Several studies find that consumer satisfaction is also significantly influenced by Airbnb service (Ju et al., 2019; Tussyadiah and Zach, 2017), facilities (e.g., indoor environmental quality) (Villeneuve and O'Brien, 2020), location, feeling welcome (Tussyadiah and Zach, 2017), user characteristics (Moro et al., 2019), perceived value (Jiang et al., 2019), and face-to-face interaction between guests and host (Moon et al., 2019a).

A study by Zhu et al. (2019) shows that host verification information, communication, renting policy, information about the environment, space offered, price, and experience influence guest satisfaction. Although the host and guest interaction may generate satisfaction, in some situations it may increase the likelihood of complaints as well (Lu et al., 2020). Finally, ratings on experiences, location, and products/services significantly influence the customers' overall satisfaction (Luo and Tang, 2019).

d. Loyalty

Loyalty, or the continuous consumption behavior of using a sharing accommodation, such as Airbnb, is influenced by several factors. According to Huarng and Yu (2019), customer loyalty is directly influenced by customer satisfaction, while customer satisfaction is influenced by network platform service quality, lodging service quality, and experience. Their results are consistent with (Priporas et al., 2017b). Interestingly, a study by Huang et al. (2020) demonstrates that the host's service and the perceived value that could drive satisfaction can also cause discontinuance.

As for loyalty and service quality, customers who use P2P platforms like Airbnb feel that the emotional perception they gain from these platforms is the most substantial contributor to loyalty (Clauss et al., 2019). This finding is supported by Yang et al. (2017). According to their research, safety benefits, a new type of relational benefit, also significantly affect commitment in this contex. Commitment acts as a mediator among confidence, social and safety benefits, and customer loyalty (Yang et al., 2017). As a dimension of relationship quality, commitment also affects loyalty. The two types of commitment, affective commitment and calculative commitment, play a significant role in enhancing loyalty to Airbnb (Kim and Kim, 2020). Other studies related to loyalty show that attitude (Yang and Ahn, 2016), service quality, and social authenticity significantly influence Airbnb loyalty (Lalicic and Weismayer, 2018). Authenticity has also been examined with social interaction and both significantly influence post-failure loyalty. The three components of authenticity (brand authenticity, existential authenticity, and intrapersonal authenticity) can enhance loyalty through the mediation of brand love (Mody and Hanks, 2020).

Regarding loyalty to Airbnb, one conceptual study offers the concept of a “home feeling,” divided into two areas, a home feature and a home meaning. The construct is found to affect three types of loyalty outcomes (cognitive, affective, and conative) (Zhu et al., 2019a). The study also finds that a more specific type of loyalty (e.g., brand loyalty) significantly influences the authenticity offered by Airbnb among the guest, the site, and others in the environment (see Table 4).

**Table 4:** Factors affecting Airbnb consumers.

Dependent Variable	Independent Variable	Mediating Variable	Author
a. Purchase intention	Host's photo	Host's attractiveness	Ert et al. (2016)
	Host's photo	Visual-based trust	
	Gender		
	Hedonic motivation		Lin et al. (2017)
	Price value		
	Habit		
	Facilitating condition		
	Social presence	Trust	Ye et al. (2019)
		Social connection	
		Usefulness	
		Ease of use	
	Experience economy	Extraordinary outcome	Mody et al. (2017)
		Memorability	
	Socio-economic orientation and price sensitivity		Pappas (2017)
	Benefits, risk, and trust		
	Insecurity/distrust	Attitude	So et al. (2018)
	Rating volume		Chen and Chang (2018)
	Information quality	Satisfaction	
	Enjoyment	Attitude	Sung et al. (2018)
	Network effect		
	Level of involvement		Lee and Kim (2018)
	Subjective norm		Amaro et al. (2018)
	The desire for unique accommodation		
	Attitude		
	Economic benefit		
	Price value		Nathan et al. (2020)
	Social influence		
	Financial Risk		Yi et al. (2020)
	Privacy Risk		
	Perceived Risk	Attitude	Lee (2020)
	Perceived Benefit		
	Subjective norm		Ma et al. (2020)
	Perceived Behavioral Control		
	Trust	Attitude	Chatterjee et al. (2019)
	Perceived authenticity		
	Social norms		
	Effort expectancy		
	Price sensitivity		
	Airbnb's reputation	Guests' attachment	Tiamiyu et al. (2020)
	Guests' unique experience		
	Perceived Value		Tran and Filimonau (2020)
	Functional aspects		
	Guest demographics		Lu and Tabari (2019); Mody et al. (2017); Tussyadiah and Pesonen (2018)
	Guest psychological characteristics		Chiappa et al. (2020); Gupta et al. (2019); Tsourgiannis and Valsamidis (2019)
	Guest geographical characteristics		Guttentag et al. (2018); Latif et al. (2019); Volgger et al. (2019)
	Guest travel characteristics		Lin (2018); Tussyadiah and Park (2018); Volgger et al. (2019); Yang et al. (2019); Young et al. (2017)
b. Repurchase intention	Trust in the company		Liang et al. (2018))
	Trust in host		
	Transaction-based satisfaction		
	Perceived value	Attitude	Mao and Lyu (2017)
	Perceived risk	Subjective norm	
	Unique experience expectation		
	Familiarity		
	Electronic word-of-mouth		
	Perceived usefulness	Attitudes	Wang and Jeong (2018)
	Trust	Satisfaction	
	Perceived authenticity	Satisfaction	Birinci et al. (2018)
	Safety and security risk		
	Perceived authenticity	Perceived value	Liang et al. (2018)
	Electronic word-of-mouth	Perceived risk	
	Price sensitivity		
	Customer experience		Li et al. (2019)
	Service failure	Customer satisfaction	Chen et al., 2019a, Chen et al., 2019b
	Recovery strategies		
c. Satisfaction	Facilities		Xu et al., 2019a, Xu et al., 2019b
	Home experience		
	Host attitude		
	Service		Tussyadiah and Zach (2017)
	Facilities		
	Location		
	Feeling welcome		
	The comfort of a home		
	Functional value		Sthapit et al. (2019)
	Social value		
	Co-creation		
	∖Information overload		
d. Loyalty	Network platform service quality	Satisfaction	Huarng and Yu (2019); Priporas et al. (2017b)
	Lodging service quality		
	Experience		
	Emotional perception		Clauss et al. (2019)
	Confidence	Commitment	Yang et al. (2017)
	Social and safety benefits		
	Service quality		Lalicic and Weismayer (2018)
	Social authenticity appeal		
	Authenticity		Shuqair et al. (2019)
	Social interaction		
	Authenticity	Brand love	Mody et al. (2019a)
		Well-being	
		Memorability	

Previous studies find that guests’ demographics, psychology, geography, and travel characteristic significantly influence their intention to use Airbnb services. The literature discusses the guest demographic characteristics of Airbnb as well. Airbnb users are mostly well educated, younger and married with children (Mody et al., 2017). However, in terms of income, they may either have higher (Mody et al., 2017), or lower income than non-users (Lu and Tabari, 2019). Like Mody et al. (2017), Tussadiyah and Pesonen (2018) support that Airbnb users tend to be well educated.

Apart from demographic characteristics, psychological characteristics, such as decision-making styles (Chen et al., 2019a, Chen et al., 2019b) national cultural values (Gupta et al., 2019), personality (Tsourgiannis and Valsamidis, 2019), and motivations (Chiappa et al., 2020), are shown to be differentiating factors that influence Airbnb customer behavior.

Geographical characteristics of the guests, such as country of origin, also influence the likelihood of using Airbnb. Those who come from an internet-affinity (high-speed internet connection) countries, such as Singapore, tend to stay in Airbnb accommodations (Volgger et al., 2019). A study by Guttentag et al. (2018) shows that customers in the US and Canada are motivated to stay in Airbnb accommodation based on their interactions, the home benefit, the novelty, the sharing economy ethos, and local authenticity offerings. Similarly, a study by Latif et al. (2019) in Malaysia shows the effect of authenticity, along with price value, social interaction, and home benefit on Airbnb customer decision-making.

Customers' travel style, purpose, and length of the trip also are factors that influence consumer preferences to choose between P2P accommodations and hotels. Group and family travelers tend to choose Airbnb accommodations compared with solo travelers because of the price (value for money), quality of accommodation, unique experience, and location (Lin, 2018; Volgger et al., 2019; Yang et al., 2019). For leisure, consumers prefer to stay in P2P accommodations because of the price, location, party size, dwelling size, and trip length (Tussyadiah and Park, 2018; Volgger et al., 2019; Yang et al., 2019; Young et al., 2017). While for business travel, consumers prefer to stay at a hotel due to location, safety, security, price, and assurance of the delivery of expected services (Tussyadiah and Park, 2018; Volgger et al., 2019; Yang et al., 2019; Young et al., 2017). In terms of trip length, a hotel is preferred for trips within seven days, while for longer trips, Airbnb is preferred (Yang et al., 2019).

In addition to the demographic, psychological, geographical, and travel characteristics, studies have compared the perceptions and motivations of Airbnb users and non-users (Huang et al., 2019). Airbnb users tend to prioritize price while non-users emphasize service (Poon and Huang, 2017). Moreover, Volgger et al. (2019) find that user versus non-user differences in terms of overall spending behavior should also be considered.

The empirical study by Varma et al. (2016)finds that non-users of Airbnb are unaware of its existence as an alternative. Interestingly, among both Airbnb users and non-users, there is not much concern about the safety and security of Airbnb lodgings (Varma et al., 2016). Guttentag and Smith (2017) find that customers perceive Airbnb service attributes as significantly outperforming budget hotels but significantly underperforming upscale hotels. In terms of experience and satisfaction, customers give Airbnb higher ratings than they give to hotels (Huarng and Yu, 2019).

e. Other variables

In addition to the research related to purchase intention, repurchase intention, satisfaction, and loyalty, scholars have examined other factors related to guest behavior surrounding Airbnb use. For example, Wu and Shen (2018) specifically examine types of trust in the Airbnb context. They conclude that institutional trust positively affects product trust and interpersonal trust, while product trust positively affects interpersonal trust. Another study finds that persuasive cues, such as credibility, emotional bonding, and accommodation characteristics, have a significant effect in establishing trust (Yang et al., 2018). Other research has shown that customer's trust in Airbnb is also influenced by host attributes and location (Cheng et al., 2019), references (Costa et al., 2017), the number of reviews (Zhang et al., 2018), and the ranking of the accommodation (Costa et al., 2020). Yang et al. (2018) examine cognitive and affective features contributing to creating users' trust in Airbnb. Their results show that three cognitive features (security and privacy, information technology quality, Airbnb traits) and affective features (reputation, interaction, familiarity) also contribute significantly to trust development.

Another topic discussed in the academic journals relates to the online review of the customer experience (Bae et al., 2017). Compared with the traditional economy, reviews in the sharing economy are more favorable (Santos et al., 2020). The Airbnb sharing economy platform generates three types of social contacts that can enhance the guest experience: guest–host, guest–guest, and guest–community (Lin et al., 2019). The involvement of these parties on the online platform can resolve complaints (Moon et al., 2019b).

Although negative reviews are submitted along with positive reviews (Bridges and Vásquez, 2018), negative reviews on Airbnb are more credible (Zhang, 2019). It is important to note that a review of an Airbnb site is not one sided; the guest can review the host, and the host can also review the guest (Liang et al., 2019). This mechanism drives guests toward socially desirable behaviors when they share information about their transactions (Newlands et al., 2019). Although some customers may have poor experiences, they do not necessarily share these poor experiences through online reviews (Meijerink and Schoenmakers, 2020), as these guests may feel that negative reviews may be harmful to their profiles (Bulchand-Gidumal and Melián-González, 2020).

In terms of negative reviews, one study finds that less social distance and greater empathy from a guest may reduce negative reviews (Pera et al., 2019). Face-to-face interaction between the host and guest can also reduce negative comments by guests (Baute-Díaz et al., 2019). Online negative reviews about poor customer service and the hosts' unpleasant behavior are two factors that lead to distrust (Sthapit and Björk, 2019). However, customers do not share negative experiences when there is a good guest–host relationship (Osman et al., 2019). Additionally, there are differences in how users express their experiences in online reviews (Hernández-López, 2019). When they feel disappointed, they write more formal and objective online reviews. By contrast, when they feel satisfied, they post very friendly online reviews.

In terms of topics, Sutherland and Kiatkawsin (2020) identify some issues in online reviews in New York. Their topics revolve around the accommodation experience (e.g., WOM, complaints), location (e.g., navigation information), accommodation unit (e.g., security, cleanliness), and management (e.g., friendliness, empathy). The reviews also affect the hosting price and other neighboring hosts, as reviews are considered quality indicators (Lawani et al., 2019). It is worth noting that the Airbnb guests’ mindset regarding what is considered good quality accommodations changes over five years and during different seasons (Lee et al., 2019a, Lee et al., 2019b).

Another topic discussed in the literature relates to disabled customers (Randle and Dolnicar, 2019). According to Randle and Dolnicar (2019), Airbnb properties now offer more accommodation for the disabled, specifically removing physical barriers versus just information barriers related to accessibility.

Concern about cross-cultural issues are also observed in the literature. Cross-cultural studies have examined whether there is a convergence or divergence of dimensions of Airbnb. as seen by Indians, Portuguese, and Americans. The results show a convergence in how those three cultures perceive Airbnb (Brochado et al., 2017). Another cross-cultural study examines how the West perceives Chinese guests; the results show that cultural differences exist and may result in guest–host challenges and disputes (Zhu et al., 2019b). When traveling with a companion to China, American consumers prefer to stay at an outgroup host, while Chinese consumers prefer an ingroup host regardless of whether they are traveling alone or with a companion (Wang et al., 2019).

Finally, the literature discusses other guest behavior. One factor that can encourage guest cleaning behavior is social presence (Ranson and Guttentag, 2019). Camilleri and Neuhofer (2017) propose a theoretical framework that portrays the host–guest social interaction in co-creation. Using a theoretical framework of co-creation, Johnson and Neuhofer (2017) explain how guests actively engage in value co-creation in terms of operant resources, value co-creation practice, and co-creation outcomes. Research from the customer perspective also examines the importance of convenience and assurance factors in service quality perceptions of Airbnb (Priporas et al., 2017a). Flexibility, ease of access to specific tourist sites, and efficient problem resolution are all considered necessary by customers.

#### 4.3.3 Communities/neighbors

Before we discuss the impact of Airbnb on communities, the characteristics of the cities where Airbnb is present are also important to discuss (see Table 5). The characteristics of cities can be assessed through three dimensions: geographic, social, and economic. Geographically, the Airbnb supply is significantly and negatively influenced by distance to the city center (Benítez-Aurioles, 2018b; Gibbs et al., 2018a; Gutiérrez et al., 2017; Ioannides et al., 2018; Jiao and Bai, 2020a; Quattrone et al., 2018; Rubino et al., 2020; Sarkar et al., 2013; Schäfer and Braun, 2016; Xu et al., 2019a), and positively influenced by city size (Adamiak, 2018), proximity to points of interest (POI) (Contu et al., 2019b; Dudás et al., 2017b; Quattrone et al., 2018; Sarkar et al., 2013; Tussyadiah and Zach, 2017), and infrastructure and transportation availability (Quattrone et al., 2018). While some scholars find a concentration of Airbnb listings in areas where hotel supply is significant (Domènech et al., 2019), others find that the availability of hotels has no impact on the presence of Airbnb listings (Quattrone et al., 2018). Based on POI or geographic features that might be useful or interesting (Quattrone et al., 2018), Airbnb locations are concentrated mostly in locations near: eating and drinking, attractions, retail, sports and entertainment, pubs, town halls, post-offices, and beaches (Dudás et al., 2017b; Quattrone et al., 2018; Sarkar et al., 2013). The number of POI also influences the price of Airbnb listings (Jiang and Yin, 2020).

**Table 5:** Airbnb city characteristics.

Characteristic	Indicators	Author
Geographic	Distance to center	Benítez-Aurioles (2018b); Dudás et al. (2017a); Gibbs et al. (2018a); Gutiérrez et al. (2017); Ioannides et al. (2018); Jiao and Bai (2020a); Quattrone et al. (2018); Rubino et al. (2020); Sarkar et al. (2013); Schäfer and Braun (2016); Xu et al. (2019a)
	Points of interest	
	Infrastructure and transport (e.g., number of bus stops)	
	Hotel presence	
Social	Population density	Alizadeh et al. (2018); Dudás et al. (2017b); Eugenio-Martin et al. (2019); Lagonigro et al. (2020); Quattrone et al. (2018); Sarkar et al. (2013)
	Young population	
	Children and adolescence presence	
	Talented and creative class (Bohemian index)	
	Race/diversity	
	Talent index	
	Asian ethnicity	
Economic	Number of housing units	Dudás et al. (2017b); Quattrone et al. (2018); Sarkar et al. (2013)
	Employment rate	
	Poverty	
	Mortgage	
	Household value	
	Income	

Based on social indicators, the Airbnb supply in specific locations is influenced by population size, number of tourist visits, family income, education level, dwelling size, number of young people, and the talented and creative class in the area (Alizadeh et al., 2018; Dudás et al., 2017b; Eugenio-Martin et al., 2019; Lagonigro et al., 2020; Quattrone et al., 2018; Sarkar et al., 2013); (see Table 5).

Based on economic indicators, the level of Airbnb supply in certain areas is influenced by the number of housing units, employment rate, poverty, mortgage values, household values, and income influence (Dudás et al., 2017b; Quattrone et al., 2018; Sarkar et al., 2013).

What about society's perception of Airbnb's impact on the community? Some research shows that residents have positive rather than negative perceptions of Airbnb (Dogru et al., 2019; Jordan and Moore, 2018; Yeager et al., 2020). Resident perceptions are that Airbnb enables them to interact with tourists, reduces loneliness, promotes solidarity, preserves the natural environment, and offers more business and job opportunities (Balampanidis et al., 2019; Contu et al., 2019b; Farmaki and Stergiou, 2019; Grimmer and Vorobjovas-Pinta, 2019; Jordan and Moore, 2018; Petruzzi et al., 2020; Suess et al., 2020; von der Heidt et al., 2019). In addition, Airbnb also promotes the physical rehabilitation of private buildings and city “beautification” projects (Chamusca et al., 2019), advocating for the emergence of hospitality micro-entrepreneurs (Wyman et al., 2020).

By contrast, other research argues that collaborative accommodation has an uneven effect on society; it introduces aggravating effects instead of leveling out demographic, economic, and social inequalities (Dredge and Gyimóthy, 2015). In terms of externalities, among the problems associated with Airbnb are digital discrimination, gentrification, increasing residential rental prices, domination of housing ownership, zoning/neighborhood quality, employment, trash, noise, water scarcity, waste management issues, open-air parties, annoyances, disputes, hostility, tourism phobia, prostitution, and drug issues (Amore et al., 2020; Balampanidis et al., 2019; Brauckmann, 2017; Chamusca et al., 2019, 2019; Chica-Olmo et al., 2020; Edelman et al., 2017; Gant, 2016; Garcia-Ayllon, 2018; Gil and Sequera, 2020; González-Pérez, 2020; Gurran and Phibbs, 2017; Horn and Merante, 2017; Jiao and Bai, 2020b; Jordan and Moore, 2018; Lima, 2019; Manning et al., 2018; Martin-Fuentes et al., 2018; Nieuwland and van Melik, 2018; Robertson et al., 2020; Rodríguez-Pérez de Arenaza et al., 2019; Roelofsen, 2018; Schäfer and Braun, 2016; Smith et al., 2018; Spangler, 2020; Stabrowski, 2017; Stergiou and Farmaki, 2019; von der Heidt et al., 2019; Wachsmuth and Weisler, 2018; Wyman et al., 2020; Xu et al., 2019b; Yrigoy, 2019). The most severe threats posed by the presence of Airbnb are changes in local culture including the threat of losing local authenticity and traditions (Petruzzi et al., 2020). Few journal articles investigate the prostitution and drug issues raised by Manning et al. (2018). However, posts on a popular website confirm that some Airbnb properties have been rented and turned into pop-up brothels (Bbc.com, 2017; Woods, 2017). The summary of the impacts of Airbnb are presented in Table 6.

**Table 6:** Impacts of Airbnb on communities.

Impacts	Types	Authors
Positive	Social ties creation	Balampanidis et al. (2019); Chamusca et al. (2019); Contu et al. (2019b); Farmaki and Stergiou (2019); Grimmer and Vorobjovas-Pinta (2019); Jordan and Moore (2018); Petruzzi et al. (2020); Suess et al. (2020); von der Heidt et al. (2019); Wyman et al. (2020).
	Natural environment preservation	
	Job and business opportunities	
	Fewer greenhouse gas emissions	
	Job employment	
Negative	Competition for public resources (parking)	Amore et al. (2020); Balampanidis et al. (2019); Brauckmann (2017); Chamusca et al. (2019); Chica-Olmo et al. (2020); Edelman et al. (2017); Freytag and Bauder (2018); Gant (2016); Garcia-Ayllon (2018); Gil and Sequera (2020); González-Pérez (2020); Gurran and Phibbs (2017); Horn and Merante (2017); Jiao and Bai (2020b); Jordan and Moore (2018); Lima (2019); Manning et al. (2018); Martin-Fuentes et al. (2018); Nieuwland and van Melik (2018); Petruzzi et al. (2020); Robertson et al. (2020); Rodríguez-Pérez de Arenaza et al. (2019); Roelofsen (2018); Schäfer and Braun (2016); Smith et al. (2018); Spangler (2020); Stabrowski (2017); Stergiou and Farmaki (2019); von der Heidt et al. (2019); Wachsmuth and Weisler (2018); Wyman et al. (2020); Xu et al. (2019b); Yrigoy (2019)
	Digital discrimination	
	Rising housing rental prices	
	Gentrification	
	Water scarcity	
	Waste management issues	
	Prostitution	
	Losing authenticity and traditions/Change in local culture	

#### 4.3.4 Competitors

Airbnb has had a disruptive impact on the tourism industry in general and traditional hotels in particular. Its distinctive business model has changed the nature of competition by serving a niche market neglected or unserved by traditional accommodation services (Dolnicar, 2019; Gunter and Önder, 2018; Koh and King, 2017; O’ Regan and Choe, 2017; Stabrowski, 2017). Scholars and hoteliers have offered mixed opinions regarding the impact of Airbnb on the traditional hotel industry. Some argue that Airbnb's listings do not affect hotel revenue (Aznar et al., 2017; Blal et al., 2018; Hong Choi et al., 2015; Mhlanga, 2019; Varma et al., 2016). In other words, Airbnb is not a competitor of a hotel, as it targets a different segment of tourists (Ginindza and Tichaawa, 2019; Heo et al., 2019; Sainaghi and Baggio, 2020; Yang and Mao, 2020). While hotels target business segments, Airbnb accommodates a leisure segment (Sainaghi and Baggio, 2020). In fact, some find that the presence of Airbnb has a positive impact on the entire tourism industry, as visitors who stay in Airbnb accommodations tend to spend more time in tourist destinations (Strømmen-Bakhtiar and Vinogradov, 2019).

By contrast, other scholars find that Airbnb partially and negatively influences demand, occupancy, the average daily rate (ADR) of lodging and revenue per available room in traditional hotels (RevPAR) (Benítez-Aurioles, 2019; Boros et al., 2018; Dogru et al., 2019, 2020a, 2020b; Kwok and Xie, 2019; Li and Srinivasan, 2018; Manning et al., 2018; Oskam and Boswijk, 2016; Xie and Kwok, 2017; Zervas et al., 2017). Notably, low-end hotels and other lodgings such as homestays and inns, are the enterprises most affected by Airbnb's presence (Varma et al., 2016).

Various factors explain why Airbnb can successfully compete with the traditional lodging industry. First, Airbnb bypasses brand equity, which has long been regarded as a critical success factor in the accommodation sector by creating brand loyalty and trust from customers (Salvioni, 2016). By contrast, the Airbnb platform has introduced the rating and ranking concept and direct price comparability while fostering a critical judgement culture (Gössling et al., 2019). Second, Airbnb sites are predominantly around the central tourist spot in contrast to many hotels (Gutiérrez et al., 2017). Third, as an enterprise, Airbnb has few assets in comparison with traditional hotels, as the assets are owned by its hosts. Although, traditional hotels can outperform Airbnb in terms of speed of market expansion, as Airbnb has had difficulty in attracting new investments in new locations (Manning et al., 2018). Fourth, Airbnb has the benefit of only operating based on consumer ratings. By contrast, traditional lodging operates under government regulation as well as consumer ratings (Manning et al., 2018). Fifth, Airbnb allows customers who are more adventurous to experience the local culture by living like a local (Forgacs and Dimanche, 2016). Sixth, Airbnb offers a more user-friendly website compared with most traditional hotels (Forgacs and Dimanche, 2016). Seventh, Airbnb generally provides more flexibility to travelers, such as the ability to travel with pets (Zhang et al., 2019a, Zhang et al., 2019b). Eighth, Airbnb options are perceived as more affordable than hotels for accommodating more guests (Gunter and Önder, 2018; Önder et al., 2019). Finally, Airbnb offers so-called network relationality through temporary belongingness, a priori empathy, technology as a bridge to face-to-face interactions, and relational spaces (Marques and Gondim Matos, 2019).

Airbnb is generally expected to successfully compete with budget hotels/motels while losing ground to upscale hotels (Guttentag and Smith, 2017). Scholars indicate that to compete effectively with Airbnb, traditional hotels need not lower their prices, as it is not the relative price that drives customers to choose an Airbnb over a traditional hotel, but rather the overall trip value (Yang et al., 2019). Instead, Forgacs and Dimanche (2016) suggest that traditional hotels should learn from Airbnb's success by providing friendlier websites and promising authentic local experiences. They should also adapt their strategy based on the hotel's location, the average value of long-term rental contracts, and the sharing accommodation density (Aznar et al., 2019). Various negative impacts of Airbnb can also help traditional hotels highlight their benefits over Airbnb, such as higher safety, security, asset protection, and service professionalism (Forgacs and Dimanche, 2016). Ultimately, the hotel industry can lobby the government for a regulatory response, as a strategic response to Airbnb competition (Alrawadieh et al., 2020).

#### 4.3.5 Employees

The key activities carried out by Airbnb center on platform management, which consists of selling and generic administrative activities and operational cyclical activities. Thus, to deliver value, Airbnb has employees as its stakeholders. Munkøe (2017) questioned whether the Airbnb landlord should be considered an employee. If Airbnb landlords are considered Airbnb employees, they should receive remuneration, paid leave, and certain working conditions (Munkøe, 2017). As they do not receive any of these benefits, Airbnb landlords can only be considered independent contractors, not employees (Munkøe, 2017). In short, based on the review, no study discussed Airbnb employees as stakeholders. The limited number of articles on Airbnb employees is due to the fact that Airbnb falls under the sharing economy category. By contrast, many academic articles have been published on employees in the context of the Gig economy. Although the sharing economy has many similarities with the Gig economy, it is understandable that the latter focuses more on its economy, which is characterized by flexible and temporary jobs (Duggan et al., 2020; Howcroft and Bergvall-Kåreborn, 2019).

#### 4.3.6 Government/regulators

The government stakeholder relationship is crucial as the government drives the public policy process. Government is the only entity that has the legitimacy to speak on behalf of society as a whole. It can change the way sharing economy accommodations are governed, keep the market functioning, and infuse moral vision into the market (Buchholz and Rosenthal, 2004). Despite its importance, only 27 studies discuss the government's role in the context of Airbnb. In our review, the literature on Airbnb can be categorized into two groups: articles discussing community issues related to Airbnb and articles discussing suggested regulatory framework.

The first issue subject to regulation relates to discrimination. For example, the government of California signed an agreement with Airbnb that allows the California Department of Fair Employment and Housing to check whether Airbnb hosts in the region engage in discrimination (Piracha et al., 2018). Apart from discrimination based on race, there is also discrimination against disabled guests. Although Airbnb may provide an accessible room for disabled guests, government intervention is needed to ensure that facilities are available in large quantities for disabled guests (Boxall et al., 2018).

Safety is also an issue that the government is concerned with. Airbnb properties need to meet basic standards for the health and safety of the guests and need general liability insurance to cover guest health and safety (Chen et al., 2020). Short-term and subletting issues are also a concern in Airbnb rentals. At times, tenants sublet and rent a room through Airbnb (Schäfer and Braun, 2016). This facilitates the redistribution of wealth from the landlord to the tenant but encroaches on public preferences for the legal protection of property used personally or intimately (Stern, 2019). To address this, the Airbnb contractual relationship specifies that Airbnb pays the rent to the host only after the guests report that the accommodation meets their expectations; this makes it safer for Airbnb guests to use Airbnb services (Munkøe, 2017).

Another issue involving online sharing accommodation practices relates to land use planning. The sharing accommodation platform has blurred residential and tourist areas due to difficulties in monitoring these online operations. As part of the gentrification/tourist process, the switching of properties in the long-term rental market to the touristic (Airbnb) market reduces the supply of long-term properties, which drives up prices (Garcia-Ayllon, 2018; Guttentag, 2019). This process also affects the commercial life of the city, where traditional commerce moves out and convenience and souvenirs shops move in. These problems may result in neighborhood complaints, speculation in home prices, and price bubbles (Chen et al., 2020; Gurran and Phibbs, 2017; Zou, 2020). Therefore, scholars recommend that the government reviews local planning regulations (Zou, 2020).

The government should also pay attention to the kind of tourism it has (city, nature-based vs. sun and beach destinations) when developing policies, as each type has a different demand and supply characteristic (Eugenio-Martin et al., 2019). In a city and nature-based destination, the supply of Airbnb listings tends to meet tourist spatial distribution better than that of the established traditional accommodations. By contrast, for sun and beach destinations, the demand is better matched by the established hotel supply.

It is worth noting that Airbnb regulation can only be addressed after the government clearly defines whether Airbnb hosts and landlords are considered businesses or private individuals (Belotti, 2019; Munkøe, 2017). If Airbnb hosts are considered as a business, then numerous administrative and regulatory requirements need to be met. Many scholars have proposed different regulatory frameworks (Avdimiotis and Poulaki, 2019; Biber et al., 2017; Chen et al., 2020; Frenken et al., 2019; Llop, 2017; Strømmen-Bakhtiar and Vinogradov, 2019; Tham, 2016) (see Table 7).

**Table 7:** Proposed regulatory framework.

Topics	Types	Authors
Regulatory framework	Integrated vs. fragmented	Tham (2016)
	Block/free pass/old reg/new reg	Biber et al. (2017)
	License/Record Keeping/Standard Requirement/Host stay/Restrict eviction/Restrict conversion/Cap/Neighborhood protection/Tax	Chen et al. (2020)
	Involvement or development/moderate level of restriction/Law or limit Airbnb operation	Avdimiotis and Poulaki (2019)
	Enforce/new regulation/deregulation/toleration	Frenken et al. (2019)
	Suspension/monitoring, control and penalties/regulation	Llop (2017)

Tham (2016) classifies the regulatory framework into two types: (1) integrated and (2) fragmented. Singapore applies the integrated law to regulate Airbnb to enhance its smart-house status (Tham, 2016). By contrast, Australia applies fragmented regulation, namely, each territory in Australia has a different regulatory framework for Airbnb (Tham, 2016).

Other scholars, such as Biber et al. (2017), have proposed four frameworks to guide regulators in managing policy disruption. The first is the block, where existing regulation is preserved, and new business forms are blocked (Biber et al., 2017). The second is the free pass, which allows innovation without updating regulations (Biber et al., 2017). The third is old reg, which applies the existing regulatory structure imperfectly (Biber et al., 2017). The last is new reg, where the government creates a new code following the latest business structure (Biber et al., 2017).

Chen et al. (2020) propose a regulatory framework consisting of (1) a license, which refers to the registration or permit for the property to be used as a short-term rental; (2) record-keeping, that forces the host to report the guest name, contact information, dates of stay, among other elements; (3) standard requirements, which require the hosts to provide basic standards for the health and safety of their guests; (4) host stay, which requires the hosts to stay at the property for at least a minimum number of months/years at the time of hosting sharing accommodation; (5) restriction of eviction, prohibiting units that have recently been subject to eviction from being registered as sharing accommodations; (6) restriction of conversion, which limits rentals of single-family structures constructed less than five years before the date of application for a sharing accommodation; (7) caps, which set a cap on the number of rental nights per year; (8) neighborhood protection, which requires the sharing accommodation contract to include a copy of the local sound/trash/parking ordinance and provide a hotline to allow neighbors and other citizens to report non-emergency issues; and (9) taxation, which requires the sharing accommodation to pay hotel taxes.

Regarding taxation, William and Horodnic (2017) suggest classifying two policy approaches for regulating an informal sharing economy like Airbnb. The first is a direct approach, which is divided into (a) deterrents: by improving detection or sanctions such as raising penalties for not declaring income from informal sharing economy activities (Leshinsky and Schatz, 2018); and (b) an incentive approach such as providing a tax-free limit. The second approach is considered indirect as it involves, for example, conducting host and guest education. The government is also advised to provide practical guidelines regarding tax issues; for example, the Australian Taxation Office gives practical guidelines on how to record income and expense receipts (Tham, 2016).

However, the way the government regulates Airbnb does not have to involve rigid choices between particular regulatory frameworks (e.g., integrated vs. fragmented). Different jurisdictions may apply different regulations to control the sharing of accommodation (McKee, 2017). In this context, the government needs a more dynamic regulatory approach that can adapt to a changing environment (Grimmer et al., 2019).

Although some scholars find that regulation may have a negative impact on the development of short-term rentals (Furukawa and Onuki, 2019), it has not been proven that this will restrain the growth of the sharing economy in the long run (Chen et al., 2020). In particular, one study finds that the stricter the regulation is, the higher the supply in the sharing economy accommodation (Hong and Lee, 2018; Uzunca and Borlenghi, 2019). Ultimately, regulation of sharing accommodations should have a positive impact on the lower-scale hotels (Yeon et al., 2020).

### 4.4 How much power and influence does the stakeholder have?

**Figure 4:**
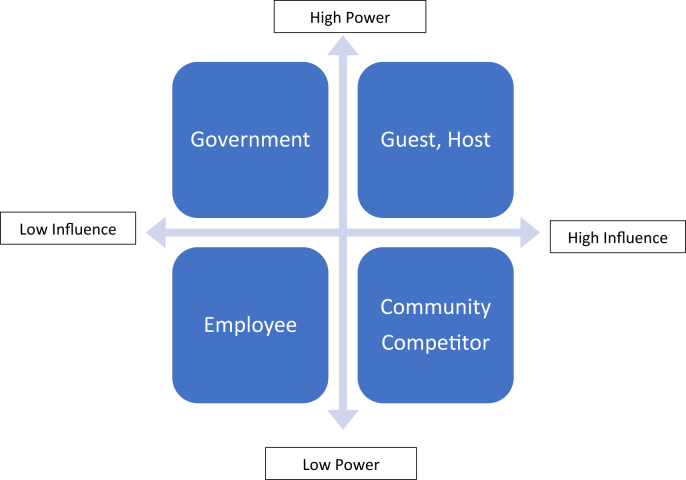
Power/influence matrix of Airbnb.

On the sharing economy platform, scholars expect that Airbnb will create empowerment that promotes equity among stakeholders (Farmaki and Kaniadakis, 2020). However, in terms of influences, the studies find that the six stakeholders (hosts, guests, communities, competitors, employees, and government) have unequal degrees of power and influence. A power/influence matrix is used to analyze the nature of these relationships and how much power and influence the Airbnb stakeholders hold over each other (see Figure 4). Power is defined as the level of authority one stakeholder has in the organization, while influence is the level of involvement the party has (Spitzeck and Hansen, 2010).

The research reviewed shows that guests are considered the most important and influential stakeholder to both Airbnb and the hosts. Without the guests, there would be no revenues for Airbnb or the hosts. These guests receive many advantages by using Airbnb, such as the benefits of staying in someone's home, cost-effective pricing, value, and the enjoyment of living like a local (Amaro et al., 2018; Guttentag et al., 2018; Mao and Lyu, 2017; So et al., 2018). However, some consumers comment that Airbnb services lack value and are low in quality (Tran and Filimonau, 2020). Although the hosts and guests can review each other, the guest reviews have a much stronger influence on the hosts, as these customer reviews determine the host's status (Gunter, 2018).

Like the guests, the hosts can be considered a powerful and influential stakeholder for Airbnb, as without them, Airbnb cannot provide its services. Reciprocally, Airbnb has a strong influence on its hosts. Airbnb acts as an important platform for the hosts as it enables them not only to receive additional income but also to learn about the guest's country of origin without leaving their home through social interaction with their guests (Farmaki and Kaniadakis, 2020). In other words, Airbnb places the hosts both on the supply and demand side of the tourism sector (Dolnicar and Talebi, 2020). Although Airbnb empowers the hosts in selecting their guests, a customer-oriented Airbnb has reduced this power. To gain superhost status on the platform, hosts are required never to reject or cancel any booking (Farmaki and Kaniadakis, 2020). Airbnb also limits the host's power to select guests to avoid discrimination issues (Farmaki and Kladou, 2020).

Based on the above, these studies confirm that both the guests and hosts are the key stakeholders in Airbnb. As Airbnb's most powerful and influential stakeholders, they lie in quadrant one. The type of relationship between Airbnb and both stakeholders is cooperative. Therefore, it makes sense that Airbnb should fully engage these stakeholders and try hard to satisfy them.

To date, the sharing economy is a highly unregulated business (Sundararajan, 2016). As explained, government in different areas applies different regulations (McKee, 2017). However, the government's power is strong, as it has the ability to change where Airbnb can and cannot operate, as happened in Australia (Grimmer et al., 2019). Therefore, Airbnb needs to pay attention to governmental concerns to ensure its satisfaction.

Despite job and business opportunities or other positive impacts from Airbnb, the community as stakeholder suffers the most as it needs to deal with any negative externalities such as gentrification and noise (Dudás et al., 2017b; Farmaki and Kladou, 2020; Gurran and Phibbs, 2017). Unfortunately, no study discusses the Airbnb policy to overcome these negative or ethical issues. Our conclusion is that the nature of the relationship of Airbnb with society is one of conflict rather than cooperation. Therefore, the community can be positioned in quadrant three as high in influence but low in power. However, the community may influence the government by taking collective action or lobbying policymakers to change policy/laws (Cheng and Foley, 2018). Therefore, the community becomes a stakeholder that should be kept informed and satisfied by Airbnb to ensure that no major issues arise.

Like the community, the competition also lies in the third quadrant as high in influence but low in power. Since the study found that Airbnb has a partially negative influence on demand, occupancy, the ADR of lodging, and RevPar room in traditional hotels (Benítez-Aurioles, 2019; Boros et al., 2018; Dogru et al., 2019, 2020a, 2020b; Kwok and Xie, 2019; Li and Srinivasan, 2018; Manning et al., 2018; Oskam and Boswijk, 2016; Xie and Kwok, 2017; Zervas et al., 2017), competitors tend to become opponents of Airbnb. Thus, competitors are stakeholders that should be kept informed and satisfied by Airbnb. However, it is important to note that currently, as of 2018, Airbnb has been welcoming boutique hotels and bed and breakfasts to list on its platform, making it possible for competitors to move from the competition to the host position (Airbnb, 2019b).

As explained, virtually no study in the review discussed Airbnb employees. Due to COVID-19 and plunging revenue, the company had to lay off 1900 workers and contract employee (Torres, 2020). Despite giving uniquely generous compensation to those laid off (Kelly, 2020), Airbnb's compensation to its contract workers was considered unsatisfactory, creating a backlash among its contract employees (Furman, 2020). We conclude that employees are the stakeholders low in power and influence over Airbnb, and Airbnb puts forth a minimum effort to satisfy them.

## 5 Discussion, conclusions, limitations, and future research directions

This paper answered the following questions: 1) who are the stakeholders of Airbnb? (2) what are their interests/concerns? and (3) how much power and influence do these stakeholders have? This review proved useful for analyzing ethical aspects and cooperation and conflict that can arise among stakeholders, as explained by Harrison and Wicks (2013).

Based on the literature review, we found that the ethical issues related to the relationships between Airbnb and its stakeholders are still being investigated. The ethical issue in the relationship between Airbnb and its hosts lies in the reduction of the power of the Airbnb host to select guests, since Airbnb creates the Superhost status, which forces the hosts to accept all guests (Farmaki and Kaniadakis, 2020). Airbnb seems to be proactive in overcoming ethical issues related to guests. For example, it signed the agreement with the California Department of Fair Employment and Housing to avoid racial discrimination against the guests (Piracha et al., 2018). Airbnb has also tried to provide more facilities for disabled guests (Boxall et al., 2018).

However, despite the positive impact of Airbnb's presence in the communities (e.g., dispersed spending in neighborhoods), it has also generated negative externalities (Balampanidis et al., 2019; Contu et al., 2019b) such as gentrification (Dudás et al., 2017b) and noise (Petruzzi et al., 2020). Unfortunately, negative or ethical issues created by Airbnb's presence in the neighborhoods need to be overcome via government intervention, as Airbnb seems to be less proactive in finding its own solutions to such negative externalities. Therefore, we conclude that the relationship between Airbnb and the community seems to be in greater conflict than that with the other stakeholders.

Regarding employees, few studies examined Airbnb from the employee's perspective even though the mass media has covered the potential ethical violation of Airbnb in the layoff of its contract workers (Furman, 2020). It appears that the ethical issue related to the industrial relations in the sharing economy has been overlooked by scholars, as no study in our review discusses Airbnb's employees.

The above discussion leads to the following future research directions. First, as there is a dearth of research on Airbnb employees, future researchers are encouraged to conduct research on the ethical issues in the relationship between Airbnb and its employees.

Second, the review indicates that the research related to hosts and customers has been conducted mostly from a positive perspective. Future researchers should examine host problems related to guests, such as dealing with bad guests, prostitution, drug problems, and violence. The review also suggests the need for more cross-cultural research on Airbnb adoption.

Third, we found mixed results regarding the impact of Airbnb not only economically and socially on the community but also on environment sustainability. Thus, comparative and comprehensive research is needed to examine both positive and negative impacts simultaneously.

Fourth, as Airbnb is still unevenly distributed or concentrated in certain areas (Benítez-Aurioles, 2018b), future research should analyze the distribution of the benefits of the platform socially (social eviction) and economically (real estate speculation). Specifically, researchers could investigate the impact of Airbnb's presence on the daily lives of residents and the conflicts that could arise between locals and tourists who stay in Airbnb sites.

Fifth, despite its disruptive impact on the hotel industry, Airbnb has started to pursue boutique hotel inventory. Therefore, future research could examine the contentious and co-dependent relationship between Airbnb and hoteliers. It is also crucial for future researchers to explore the strategies that hotel brands should take to battle back.

Sixth, from the policymaker perspective, it is important to describe whether Airbnb, as a type of collaborative economy, is in the public interest and, in this context, understand how the government defines public interest. More comparative research is also needed, including comparing the short-term rental impact in some regions or countries with a low-income population compared with that in a high-income population. A study comparing the effectiveness of different types of regulatory frameworks and their implications for stakeholder welfare would also be useful.

Seventh, only limited research on Airbnb has been longitudinal. Thus, more studies using a longitudinal approach are needed to provide an empirical basis from which policymakers can shape opinions, create policies, and implement societal changes. Future research should examine simultaneously all the actors in the co-creation process related to the service provided by Airbnb and its impact on human and non-human actors.

Eighth, the current study does not discuss the literature in terms of research methods. A future literature review could compare the different results of big data and small data samples with our results on Airbnb. Future studies could also utilize a triangulation approach, which compares qualitative versus quantitative methods to add validity to the Airbnb study conducted by So et al. (2018).

Ninth, our analysis excludes other collaborative economy accommodations, such as CouchSurfing (Geiger et al., 2018; Kim et al., 2018). Future studies could compare other collaborative economy accommodations with Airbnb.

Finally, this study was finalized during the COVID-19 pandemic. This situation has raised the question: how will the pandemic change the home-sharing landscape? Future researchers are expected to examine the impact of the COVID-19 pandemic on stakeholder interests.

## Declarations

### Author contribution statement

All authors listed have significantly contributed to the development and the writing of this article.

### Funding statement

This work was supported by 10.13039/501100006378Universitas Indonesia under the Q1Q2 Research Grant ​contract number NKB-0188/UN2/R3.1/HKP.05.00/2019.

### Data availability statement

No data was used for the research described in the article.

### Declaration of interests statement

The authors declare no conflict of interest.

### Additional information

No additional information is available for this paper.
